# Physical activity and the outcome of cognitive trajectory: a machine learning approach

**DOI:** 10.1186/s11556-024-00367-2

**Published:** 2025-01-10

**Authors:** Bettina Barisch-Fritz, Jay Shah, Jelena Krafft, Yonas E. Geda, Teresa Wu, Alexander Woll, Janina Krell-Roesch

**Affiliations:** 1 Karlsruhe Institute of Technology, Karlsruhe, Germany; 2 Arizona State University, Tempe, USA; 3 Barrow Neurological Institute, Phoenix, USA

**Keywords:** Cognitive deterioration, Alzheimer's disease, Neurodegenerative diseases, Machine learning, Physical activity interventions, Artificial intelligence analysis

## Abstract

**Background:**

Physical activity (PA) may have an impact on cognitive function. Machine learning (ML) techniques are increasingly used in dementia research, e.g., for diagnosis and risk stratification. Less is known about the value of ML for predicting cognitive decline in people with dementia (PwD). The aim of this study was to use an ML approach to identify variables associated with a multimodal PA intervention that may impact cognitive changes in PwD, i.e., by distinguishing between cognitive decliners and non-decliners.

**Methods:**

This is a secondary, exploratory analysis using data from a Randomized Controlled Trial that included a 16-week multimodal PA intervention for the intervention group (IG) and treatment as usual for the control group (CG) in nursing homes. Predictors included in the ML models were related to the intervention (e.g., adherence), physical performance (e.g., mobility, balance), and pertinent health-related variables (e.g., health status, dementia form and severity). Primary outcomes were global and domain-specific cognitive performance (i.e., attention/ executive function, language, visuospatial skills, memory) assessed by standardized tests. A Support Vector Machine model was used to perform the classification of each primary outcome into the two classes of decline and non-decline. GridSearchCV with fivefold cross-validation was used for model training, and area under the ROC curve (AUC) and accuracy were calculated to assess model performance.

**Results:**

The study sample consisted of 319 PwD (IG, *N* = 161; CG, *N* = 158). The proportion of PwD experiencing cognitive decline, in the different domains measured, ranged from 27–48% in CG, and from 23–49% in IG, with no statistically significant differences and no time*group effects. ML models showed accuracy and AUC values ranging from 40.6–75.6. The strongest predictors of cognitive decline or non-decline were performance of activities of daily living in IG and CG, and adherence and mobility in IG.

**Conclusions:**

ML models showed moderate performance, suggesting that the selected variables only had limited value for classification, with adherence and performance of activities of daily living appearing to be predictors of cognitive decline. While the study provides preliminary evidence of the potential use of ML approaches, larger studies are needed to confirm our observations and to include other variables in the prediction of cognitive decline, such as emotional health or biomarker abnormalities.

## Background

Modifiable lifestyle factors such as physical activity (PA) play an important role in quality of life in people with dementia (PwD), and may also be associated with less pronounced cognitive decline [1, 2]. An umbrella review including 11 meta-analyses found positive effects of multicomponent and single-component PA interventions on global cognition, executive function and delayed memory in PwD, but no effects on verbal fluency, attention and immediate memory [3]. Similarly, another umbrella review, which included 27 systematic reviews, reported small positive effects of mind–body or multimodal PA interventions on global cognition, with resistance training having the largest effects [4]. In contrast, a meta-analysis including 5 studies with a total of 438 participants and examining the effectiveness of multicomponent PA interventions on physical fitness, cognition, and activities of daily living (ADL) in PwD, found no effects on cognitive function [5]. Another systematic review and meta-analysis including 13 studies examined the effects of various PA interventions on cognition in a total of 869 persons with Alzheimer's disease (AD). Eight trials included in this review showed that PA can improve cognition or slow cognitive decline in AD patients, but five trials showed no effect [6]. The results of the aforementioned umbrella and systematic reviews suggest that the evidence for the effects of PA on cognition in older adults is currently low to moderate. However, there are several clinical and observational studies of high methodological quality showing a positive association between PA and a reduced risk of cognitive decline in cognitively healthy persons and those with MCI [7–9]. The remaining controversy may partly be due to the high heterogeneity of PwD regarding symptom severity, or motor and cognitive performance status [10], which in turn is associated with various challenges in delivering PA interventions or evaluating their effectiveness. Moreover, different contents of PA interventions studied may contribute to the inconsistent results [6]. Challenges also pertain to the statistical analysis of data, as participant heterogeneity may impede comparisons of mean values at baseline and follow-up between intervention and control groups, as is often done in Randomized Controlled Trials (RCT) to provide an intuitive and generally unbiased estimate of the average treatment effect of an intervention. One approach to solving this problem is to consider and adjust for covariates [11]. However, research shows that including covariates may also introduce bias, particularly in small-sample RCTs [12].

In recent years, machine learning (ML) techniques, that may add value to improve the interpretation of RCT results, are increasingly being used to improve the interpretation of RCT results [13]. ML techniques used to date have mainly included adjunctive treatment decisions, adjunctive diagnostics and risk stratification. Many automated diagnostic systems based on ML techniques have been proposed in the literature for early detection of dementia [14].

We postulate that ML techniques could also provide valuable insights about the associations between PA and cognitive performance in PwD. Albeit research showed that PA may improve various cognitive functions [3–6], it is less clear 1) which baseline variables (e.g., related to sociodemographic features, PA behavior, physical performance) are predictive of cognitive changes over time in PwD; 2) whether there are differences between global and domain-specific cognition (i.e., memory, attention/ executive function, language, visuospatial skills) with regard to a potential effect of PA; and 3) whether PA interventions may interact with baseline variables related to physical performance or other health-related factors to impact cognitive changes.

To partially address these knowledge gaps, we conducted an exploratory analysis of data from a large RCT of a 16-week multimodal PA intervention in PwD in Germany. The aim was to identify which baseline parameters related to physical performance and other health variables, as well as characteristics and features of the PA intervention, might have an impact on cognitive changes over time in PwD, distinguishing between cognitive decliners and non-decliners. To assess the effects of the PA intervention, and as a prerequisite for interpreting the ML analysis, the data were subjected to a classical intention-to-treat analysis, looking at distributions and time*group effects. The results of this study may inform the design and implementation of future PA interventions in PwD, and also provide valuable insights into the application of ML approaches when analyzing RCT data with small, heterogeneous samples and multiple covariates.

## Methods

For this exploratory ML-based analysis, we used secondary data from an RCT which was designed and conducted by our research team (blinded). Briefly, we implemented a 16-week multimodal PA intervention in 36 nursing homes in southwestern Germany. The intervention combined PA to train endurance/ cardiorespiratory fitness, muscular strength and balance, as well as cognitively stimulating exercises by utilizing ritualized program sequences. The study was funded by the Dietmar Hopp Foundation. The study was retrospectively registered in the German National Register of Clinical Trials (blinded), and was approved by the Ethics Committee of (blinded). A detailed description of the study methodology can be found in the study protocol [15]. The effects of the multimodal PA intervention on gait, motor/ physical performance and ADL performance using traditional statistical analysis have been published [16–18].

### Study design and participants

The multicenter RCT included standardized assessment of cognitive function, motor performance and ADL at baseline and post-intervention. Eligible participants were identified by staff of participating nursing homes. All participants, or their legal guardians, were informed of the content and aims of the study and gave their written consent to participate.

Prior to the study, eligible participants were allocated either to the intervention group (IG) or the control group (CG, also received the PA intervention after completion of the study) using minimization software (MinimPy0.3 [19]). We applied the following inclusion criteria for participation in the study: (1) diagnosis of primary dementia or “suspected dementia” (i.e., without a confirmed clinical diagnosis) verified by a general practitioner and / or based on ICD-10 criteria, (2) Mini Mental State Examination (MMSE) indicating mild to moderate dementia (MMSE: 10–24), (3) age > 65 years, (4) being able to walk for approx. 10 m with or without walking aids, and (5) clearance by a general practitioner. Participants with secondary dementia, other severe cognitive impairments, neurological or other severely acute diseases and / or no informed consent were excluded.

### Predictor variables for ML analysis

#### Multimodal PA intervention

Briefly, the 16-week multimodal PA intervention consisted of two sessions per week, each lasting approx. 60 min. The exercises took approx. 45 min and consisted of a combination of motor and cognitive tasks. Specifically, the motor tasks focused on muscular strength, balance, endurance/ cardiorespiratory fitness and flexibility, and were performed with varying durations and at moderate to submaximal intensities. Small training devices such as dumbbells, sandbags, skipping ropes or pool noodles were used. In addition, various cognitive tasks were combined with the motor exercises to provide cognitive stimulation such as memory (e.g., “What was the destination of the last imaginary journey?”), attention (e.g., remembering a particular sequence of numbers), language (e.g., naming animals), and executive function (e.g., responding to acoustic or visual cues). During the 16-week intervention, a progression of intensity of both motor and cognitive exercises was implemented, e.g., by increasing the number of repetitions or difficulty level for the motor and cognitive exercises, by following a predefined progression protocol and supervised by experienced instructors. For ML-based analysis, we used adherence to the intervention as predictor variable.

#### Physical performance

Before and after the 16-week PA intervention, physical performance was assessed, mainly focusing on ADL, mobility, balance, and lower extremity muscular strength and functionality. For ADL, the Barthel [20] questionnaire and the two task-related tests Physical Performance Test (PPT) [21] and Erlangen Activities of Daily Living (EADL) [22] were administered. Motor performance related to mobility was measured using the Timed-Up and Go Test (TUG) [23] and the 6-m walking test (6MWT) [24]. Balance was assessed using the Balance Score of Frailty and Injuries: Cooperative Studies of Intervention Techniques–4 (FICSIT) [25] and lower extremity muscular strength and functionality using the modified chair stand test (STS_mA_time, time for five repetitions, STS_mA_rep, amount of repetitions during 30 s) [26, 27]. For ML-based analysis, we used the three ADL tests (Barthel, EADL, PPT) and the five variables of physical performance (TUG, 6MWT, FICSIT, STS STS_mA_time, STS_mA_rep) as predictor variables.

#### Other baseline variables

Demographic variables, i.e., sex and age, as well as type and severity of dementia and number of medications were assessed using questionnaires, and body mass index (BMI, weight and height) was measured. Information on subjective general health status was collected using the Cumulative Illness Rating Scale (CIRS) [28]. For ML-based analysis, we used seven variables (sex, age, BMI, dementia form, dementia severity, medication number, CIRS severity index, and CIRS morbidity index) as predictor variables.

Detailed information on the multimodal PA intervention, as well as administrational assessments as part of the RCT can be found in the study protocol and previous publications on the RCT [15, 17, 18].

#### Outcomes for ML-based analysis

Cognitive performance was assessed before (baseline) and after (post) the PA intervention. All tests were standardized and administered under the supervision of trained test assessors.

#### Screening instrument for Global cognition

We administered MMSE [29] to screen for global cognition, and used MMSE total score for analysis. The score has a maximum of 30, with higher scores indicate better performance and a score of < 25 indicates further extensive assessment for dementia using cognitive test battery as detailed below.

#### Cognitive tests

Semantic verbal fluency was assessed using the Regensburg Word Fluency Test (RWT_animals) [30], subtest animals. We recorded the number of animals produced correctly per minute, with higher values indicating better performance. Executive function and visual-spatial function was assessed using the Clock Drawing Test (CDT) [31]. Deviations in the drawing were scored according to Shulman [31] from 1 to 6, with lower scores indicating better performance (1 = clock perfect, 2 = mild visuospatial errors, 3 = clock incorrect, 4 = moderate disorganization, 5 = severe visuospatial disorganization, 6 = no representation of the clock). We also used Trial Making Test part A (TMT-A) [32] to assess attention/ executive function and processing speed, with less time required indicating better performance (maximum time: 180 s). Verbal short-term and working memory was assessed using Digit Span forward and backward (DS_for, DS_back) [33], and we used length of highest digits correctly reproduced forwards and backwards for analysis, with higher values indicating better performance. Finally, we administered California Verbal Learning Test (CVLT) [34] to assess episodic verbal learning and memory. For analysis, we used correct repetitions of 16 nouns during long delay free recall, with higher values indicating better performance.

### Statistical analysis

All participants who met the inclusion criteria and were randomized to either the IG or CG were included in the dataset used for the ML-based analysis, with the exception of deceased participants. A multiple imputation procedure (fully conditional specification imputation method, ten imputations and ten iterations) was used to account for missing data. Several constraints were defined for multiple imputations, with cognitive performance as both outcome and predictor variable, supplemented by adherence, socio-demographic variables, and motor performance. To ensure the plausibility of the imputed data, other constraints were defined, such as minimum and maximum values according to the observed range in each variable, rounding according to the original data, 100 maximum case draws and ten maximum parameter draws.

The cognitive performance of each individual was classified as decline or non-decline by comparing the baseline values ​ with the values ​​after the 16-week PA intervention. A decrease in cognitive performance was referred to as a decline, a maintenance or an increase as a non-decline. The normal distribution of the data was checked using the Shapiro–Wilk test and corresponding plots. Differences in baseline characteristics between IG and CG and in outcome variables between cognitive decliners and non-decliners of the intention-to-treat sample were compared using t-tests for continuous data and chi^2^-tests for non-parametric and/or categorical data. Differences in the distribution of cognitive decliners and non-decliners between IG and CG of the intention-to-treat sample were tested using chi^2^-tests. In addition, time*group effects were calculated using two-factor ANOVA.

For the ML analysis, the cognitive variables (MMSE, RWT_animals, CDT, TMT, DS_for, DS_back, CVLT) were defined as outcome variables and health and demographic variables (sex, age, BMI, dementia form, dementia severity, medication number, CIRS severity index, and CIRS morbidity index), ADL (Barthel, EADL, PPT), and physical performance variables (TUG, 6MWT, FICSIT, STS STS_mA_time, STS_mA_rep) as predictor variables. All predictor variables were included in the ML model. Support Vector Machine (SVM) is one of the most commonly used machine learning models to classify data [35]. An SVM model was used to classify each primary outcome into two classes: (1) Decline, meaning that post-intervention scores were lower than baseline scores, and (2) Non-decline, meaning that post-intervention were equal to or greater than baseline scores. This was not the case for TMT, where less or the same time means no decrease. Therefore, for this variable, the calculation was done in reverse (pre-post) in order to be able to interpret it in the same way. It is known that the choice of hyperparameters used to train an SVM model, such as regularization parameters or kernels, can greatly impact model performance [36]. To tackle this, we use fivefold cross-validation with grid search "GridSearchCV" technique [37] for finding the optimal parameter configuration from a given set of parameters in a grid (see Table 1). Data was split in a ratio of 80:20 for training and held-out testing sets, respectively, while maintaining a similar distribution of samples with decline and non-decline in primary outcomes. GridSearchCV performs a fivefold cross-validation on the training data for hyperparameter optimization. The best set of parameters is selected using the area under the ROC curve (AUC) as a validation metric. Using this optimal set of hyperparameters, we re-train the model on the entire training set and report results on the held-out test set. This entire pipeline with the SVM model and GridSearchCV were run separately for the intervention and control groups. The performance was evaluated using AUC and the trained model's accuracy. The code supporting this study is open-source and available at GitHub [https://github.com/jaygshah/PA-CognitionML].

**Table 1:** Grid of parameters used to find best model fit using support vector machine model and grid search cross-validation technique

Machine Learning Model	Hyperparameters	Values
Support Vector Machine	Kernel	linear, poly, rbf, sigmoid
	C – regularization	0.1, 1, 2, 3, 4, 5, 10, 100
	Gamma	scale, auto
	Degree	2, 3, 5, 10

**Fig. 1:**
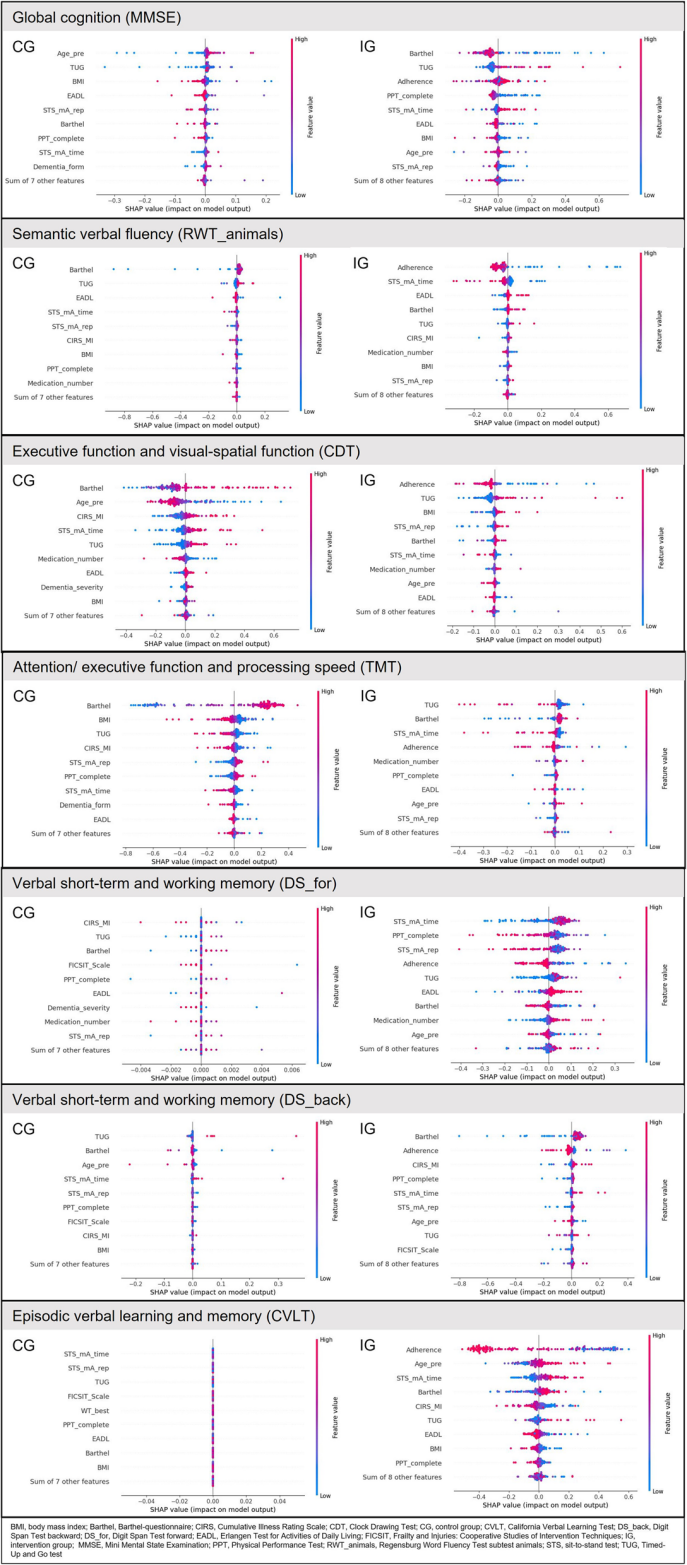
Classification of decline vs. non-decline in the cognitive outcome variables, presented by SHAP plots for CG and IG. Refer to the Statistical Analysis subsection in Methods for information on interpretation of SHAP plots

To further investigate the associations between predictor and outcome variables using the developed SVM model, we used SHapley Additive exPlanations (SHAP). SHAP is a powerful model explainability tool for understanding predictions of complex machine learning algorithms [38]. It deconstructs individual predictions into a sum of contributions from each predictor while considering their relative importance. In this study, we used SHAP’s beeswarm plot (Fig. 1) to interrogate the relative importance of predictors in a prediction and their actual relationships with outcome variables.

Interpreting SHAP plot:

1. For each predictor (input variable to the ML model), each dot represents a sample from the dataset spread horizontally along the X-axis. Samples are stacked vertically where the density of SHAP values is high. The bigger the spread, the higher the significance of the predictor in prediction.
2. The feature value color bar on the right displays the raw values of predictors and their impact on model predictions. Examining the trend of predictor variables' high (red) or low (blue) values can help understand their relationship to predicted cognitive trajectory.
3. On the left, predictors (input variables to the ML model) are listed in decreasing order of their importance to model predictions (i.e., their decreasing order of mean absolute SHAP values).
4. In Fig 1, the first plot (left) shows a SHAP plot of model predictions trained using the control group, whereas the second plot (right) shows the intervention group. Samples on the right side of the Y-axis (SHAP value > 0) were predicted as declining, whereas the ones on the left were predicted as non-declining.

## Results

### Study sample

Baseline characteristics of the study participants (IG, *N* = 161; CG, *N* = 158) are shown in Table 2. Differences in socio-demographic and other pertinent variables between the IG and CG were not statistically significant at baseline.

**Table 2:** Sample characteristics of PwD at baseline

Characteristics	Total [*n* = 319]	IG [*n* = 161]	CG [*n* = 158]	Group differences
	**M (SD)**	**M (SD)**	**M (SD)**	***P***
Age (years)	86.2 (6.1)	85.8 (6.3)	86.7 (5.8)	0.158
BMI (kg/m^2^)	28.0 (4.8)^{*n*=281}^	28.2 (4.7)^{*n*=144}^	27.8 (4.9)^{*n*=137}^	0.469
CIRS_MI	9.2 (4.7)^{*n*=186}^	9.2 (4.4)^{*n*=104}^	9.2 (5.2)^{*n*=82}^	0.909
Number of medications	7.0 (3.9)^{*n*=247}^	7.3 (3.9)^{*n*=125}^	6.7 (4.0)^{*n*=122}^	0.188
TUG	24.4 (13.7)^{*n*=238}^	23.8 (15.9)^{*n*=144}^	25.0 (12.2)^{*n*=139}^	0.477
	**n (%)**	**n (%)**	**n (%)**	***P***
Sex				0.168
Female	274 (85.9)	134 (83.2)	140 (88.6)	
Male	45 (14.1)	27 (16.8)	18 (11.4)	
Diagnosis of dementia				0.165
Yes	211 (66.1)	111 (68.9)	100 (63.3)	
No	56 (17.6)	30 (18.6)	26 (16.5)	
Unknown	52 (16.3)	20 (12.4)	32 (20.3)	
Type of dementia				0.019
AD	52 (16.3)	26 (16.1)	26 (16.5)	
Vascular dementia	46 (14.4)	32 (19.9)	14 (8.9)	
Mixed dementia	8 (2.5)	3 (1.9)	5 (3.2)	
Other	4 (1.3)	4 (2.5)	0	
Unknown	209 (65.5)	96 (59.6)	113 (71.5)	

*BMI* Body Mass Index, *CG* Control group, *CIRS_MI* Morbidity Index of the Cumulative Illness Rating Scale, *df* Degree of freedom, *IG* Intervention group, *M* Mean, *n* Number of individuals with dementia, *SD* Standard deviation, *TUG* Timed-Up and Go Test; {*n* =}, number of participants with missing information on a given variable

### Differences between cognitive decliners and non-decliners using traditional statistical analysis

The distribution of the intention-to-treat sample into decliners and non-decliners is shown in Table 3. The differences in the distribution of decliners and non-decliners are not statistically significant in IG and CG. The proportion of PwD who experienced a decline in cognitive performance during the 16-week PA intervention was 27–48% in CG and 23–49% in IG. Overall, there are no statistically significant time*group effects. The differences within IG and CG for changes in cognitive variables from pre- to post-assessment are all statistically significant.

**Table 3:** Mean differences in the intention-to-treat sample between pre and post assessments of decliners and non-decliner in IG and CG

Cognitive variables	Total sample [*n* = 319]				IG [*n* = 161]				CG [*n* = 158]			
		**Distribution of** **decliners and non-** **decliners**	Time* Group **Effects**	**Effect Size**		**Decliners**	**Non-** **Decliners**	**Differences within IG**		**Decliners**	**Non-** **Decliners**	**Differences within CG**
	**M (SE)**	***P***	***P***	**η**_**p**_^**2**^	**M (SE)**	**M (SE),** **n (%)**	**M (SE),** **n (%)**	***P***	**M (SE)**	**M (SE),** **n (%)**	**M (SE),** **n (%)**	***P***
MMSE (post–pre)	-0.3 (0.2)	0.225–0.933	0.351–0.874	0.000–0.003	-0.2 (0.3)	-3.6 (0.3), 71 (44)	2.5 (0.3), 90 (56)	< 0.001	0.1 (0.4)	-4.0 (0.4), 64 (41)	2.9 (0.3), 94 (59)	< 0.001
RWT_animals (post–pre)	-0.4 (0.2)	0.536–0.954	0.481–0.909	0.000–0.002	-0.4 (0.3)	-2.9 (0.2), 78 (49)	2.1 (0.2), 83 (51)	< 0.001	-0.4 (0.3)	-2.9 (0.3), 76 (48)	2.0 (0.3), 82 (52)	< 0.001
CDT (post–pre)	-0.1 (0.1)	0.336–0.823	0.215–0.639	0.000–0.005	-0.1 (0.1)	-1.5 (0.1), 59 (37)	0.7 (0.1), 102 (63)	< 0.001	-0.2 (0.1)	-1.5 (0.1), 52 (33)	0.5 (0.1), 106 (67)	< 0.001
TMT (pre-post)	8.4 (2.5)	0.521–0.984	0.342–0.953	0.000–0.003	7.7 (3.3)	-31.9 (4.7), 46 (29)	23.6 (3.4), 115 (71)	< 0.001	9.2 (3.5)	-33.5 (4.1), 42 (27)	24.5 (3.2), 116 (73)	< 0.001
DS_for (post–pre)	0.1 (0.1)	0.277–0.919	0.115–0.610	0.001–0.007	0.2 (0.1)	-1.4 (0.1), 45 (28)	0.7 (0.1), 116 (72)	< 0.001	0.0 (0.1)	-1.4 (0.1), 49 (31)	0.6 (0.1), 109 (69)	< 0.001
DS_back (post–pre)	0.2 (0.1)	0.035–0.531^1^	0.323–0.990	0.000–0.003	0.2 (0.1)	-1.2 (0.1), 37 (23)	0.7 (0.1), 124 (77)	< 0.001	0.2 (0.1)	-1.2 (0.1), 45 (29)	0.8 (0.1), 113 (71)	< 0.001
CVLT (post–pre)	0.8 (0.3)	0.349–0.922	0.375–0.981	0.000–0.002	0.6 (0.4)	-4.0 (0.4), 62 (39)	3.5 (0.3), 99 (61)	< 0.001	0.9 (0.4)	-3.1 (0.3), 62 (39)	3.4 (0.3), 96 (61)	< 0.001

*CDT* Clock Drawing Test, *CG* Control group, *CVLT* California Verbal Learning Test, *DS_back* Digit Span Test backward, *DS_for* Digit Span Test forward, *IG* Intervention group, *M* Mean, *MMSE* Mini Mental State Examination, *n* Number, *RWT_animals* Regensburg Word Fluency Test subtest animals, *SE* Standard error, *TMT* Trail Making Test; ^1^statistically significant difference in one iteration

### Results from the application of ML

#### Descriptions of model fit

Table 4 describes the SVM model’s performance in distinguishing samples with decline vs non-decline in cognitive outcomes on held-out test sets not included in the model training of both CG and IG. Most ML models had a weak performance (AUC less than 50) in distinguishing decline from non-decline underpinning the complex associations between predictors included here and cognitive outcomes. However, we focus on using SHAP plots to investigate these associations, despite being not strong, in the section below.

**Table 4:** Performance of the SVM models trained using grid search’s optimal set of hyperparameters on the held-out blind test-set for different cognitive outcomes

Cognitive Variables	Group	Accuracy	AUC
MMSE	CG	62.5	48.8
	IG	63.6	60.7
RWT_animals	CG	40.6	34.0
	IG	51.5	51.7
CDT	CG	59.4	50.4
	IG	60.6	40.4
TMT	CG	50.0	39.8
	IG	39.4	43.1
DS_for	CG	68.8	37.3
	IG	51.5	42.6
DS_back	CG	75.0	59.4
	IG	75.6	30.8
CVLT	CG	73.1	56.4
	IG	84.8	72.9

*AUC* Area under the ROC curve, *CDT* Clock Drawing Test, *CVLT* California Verbal Learning Test, *DS_back* Digit Span Test backward, *DS_for* Digit Span Test forward, *MMSE* Mini Mental State Examination, *RWT_animals* Regensburg Word Fluency Test subtest animals

#### Classification into decline and non-decline (SHAP plots)

The SHAP plots highlight the relative importance of each predictor and their associations to cognitive outcomes calculated from the SVM models trained to classify each sample into decline or non-decline within CG and IG groups, respectively. The SHAP plots for the outcome variables separately for CG and IG are presented in Fig. 1.

The interpretation and direction of the associations are presented in Table 5. Global cognition (MMSE), age, mobility (TUG) and BMI are the most relevant predictors of decline in CG. For IG, the most relevant predictors are ADL performance (especially Barthel, but also PPT, EADL), mobility (TUG) and adherence to the intervention. Relevant predictors in the IG for predicting decline in semantic verbal fluency (RWT) are adherence and lower extremity strength and functionality (STS). In the CG, there was too little variance to identify predictors. Predictors of decline in executive function and visuo-spatial function (CDT) are ADL performance (Barthel) and age in CG, and adherence and mobility (TUG) in IG. Relevant predictors of decline in attention/executive function and processing speed (TMT) are ADL performance (Barthel) and BMI in CG, and mobility (TUG), ADL performance (Barthel), as well as lower extremity function and strength (STS) in IG. For the prediction of decline in verbal short-term and working memory (DS_for and DS_back), the variance in the CG was too small to identify predictors here. In the IG, however, it is lower extremity strength and functionality (STS) and adherence (for DS_for), as well as ADL performance (Barthel) and adherence (for DS_back). Predictors of decline in episodic verbal learning and memory (CVLT) can also only be identified in the IG namely adherence, age, and lower extremity strength and functionality (STS).

**Table 5:** Summary interpretation of the SHAP plots and variables relevant for categorization into decliners and non-decliners

	Global cognition (MMSE)	Semantic verbal fluency (RWT_animals)	Executive function and visuo-spatial function (CDT)	Attention/executive function and processing speed (TMT)	Verbal short-term and working memory (DS)	Episodic verbal learning and memory (CVLT)
CG	Decline associated with older age, worse mobility (TUG), lower BMI, lower ADL performance (EADL), and fewer lower extremity strength and functionality (STS)	Decline associated with lower ADL performance (Barthel)	Decline associated with higher ADL performance (Barthel), CIRS, STA, and TUG scores. Non-decline linked to younger age	Decline associated with higher ADL performance (Barthel, PPT), lower extremity strength and function (STS) Non-decline associated with higher mobility (TUG), BMI, and health status (CIRS)	No conclusive analysis for DS_for and DS_back	No conclusive analysis
IG	No decline associated with higher ADL performance (Barthel) higher mobility (TUG). Adherence showed no clear trend	Decline associated with lower ADL (EADL, Barthel). Non-decline associated with higher adherence and lower limb strength and functionality (STS)	Decline associated with lower adherence, lower mobility (TUG), and higher BMI, Non-decline associated with higher adherence, higher mobility (TUG)	Decline associated with low ADL performance (Barthel Index Non-decline associated with higher mobility (TUG) lower limb strength and functionality, and adherence	DS_for: Decline is associated with lower extremity strength and functionality (STS) Non-decline associated with higher adherence, ADL performance (PPT), and higher extremity strength and functionality (STS) DS_back: Weak trends	Decline associated with older age, lower ADL performance (Barthel) lower limb strength and function (STS) Non-decline associated with higher adherence

*ADL* Activities of daily living, *BMI* Body mass index, *Barthel* Barthel-questionnaire, *CIRS* Cumulative Illness Rating Scale, *CDT* Clock Drawing Test, *CG* Control group, *CVLT* California Verbal Learning Test, *DS_back* Digit Span Test backward, *DS_for* Digit Span Test forward, *EADL* Erlangen Test for Activities of Daily Living, *IG* Intervention group, *MMSE* Mini Mental State Examination, *PPT* Physical Performance Test, *RWT_animals* Regensburg Word Fluency Test subtest animals, *STS* Sit-to-stand test, *TUG* Timed-Up and Go test

Adherence is listed as one of the top three predictors of decline for 5 of the 7 cognitive outcomes. It is even the most relevant predictor for semantic verbal fluency (RWT), executive function and visuo-spatial function (CDT), and episodic verbal learning and memory (CVLT). ADL performance, particularly the Barthel, is one of the top three predictors of 5 of the 7 cognitive outcomes in the CG. In the IG, ADL performance (but all three tests, Barthel, EADL, PPT) is also among the top three predictors for 5 of the 7 cognitive outcomes. In CG, mobility (TUG) is a significant predictor for 6 of the 7 cognitive outcomes and is among the top 3 predictors. In the IG, mobility (TUG) is among the top three predictors three times, placing it behind adherence.

## Discussion

The ML models used in this study show values for accuracy ranging from 40.6 to 75.6 and similar values for AUC. No differences were found in the number of correct classifications and in the ratio of correctly to incorrectly classified objects [39]. Overall, these values are rather low, indicating that the selected variables have only a limited ability to classify cognitive decliners and non-decliners with sufficient quality. Studies in the context of diagnostic prediction of dementia using ML approaches obtained considerably higher values for accuracy [40], thus, our results should not be over-interpreted. The discrimination power shows slight differences between IG and CG (e.g., AUC of MMSE: IG 60.7 vs. CG 48.4, of CVLT: IG 72.9 vs. CG 56.4), which allows the hypothesis that the IG has a higher predictive power than the CG. This is consistent with the assumption that the IG shows some changes (decline or no decline) due to participation in the intervention, whereas the control group should remain unchanged and the decline is more likely to be the result of ageing or the progression of neurodegenerative disease pathology [41]. However, the tendency towards low accuracy and AUC values raises the question of whether the right variables or a sufficient number of variables were used for classification. It can be assumed that cognitive decline may be better explained by other variables than the ones used in our study, e.g., neuropsychiatric symptoms, or AD biomarker abnormality. In addition, a ML-based study aimed at predicting cognitive impairment and the onset of dementia through different risk factors [42]. The investigators report that persons with high levels of emotional distress had the relatively highest risk of developing cognitive impairment and dementia, and that higher-order factors (e.g., emotional distress, subjective health) were more important for predicting cognitive impairment and dementia than narrowly defined factors (e.g., clinical and behavioral indicators). Additionally, AD biomarker analyses are also significant predictors of dementia diagnosis [43].

In the post-analyses, we focused on using SHAP to unravel relationships between PA and other pertinent variables with cognitive performance in PwD. In the IG, the most relevant predictors of decline or non-decline of cognitive performance were adherence to the PA intervention, and ADL performance (assessed by Barthel, EADL, PPT, and TUG). For 5 of the 7 cognitive domains, adherence to the PA intervention was the most relevant predictor for the classification of cognitive decline or non-decline. For the CG, the two most relevant classifiers were age and ADL performance. The variance within the cognitive domains between decliners and non-decliners tended to be higher in the IG.

When considering global cognition, our data shows that baseline ADL performance is important for differentiating decliners from non-decliners in both CG and IG, and adherence to the PA intervention is a predictor of global cognition in the IG. While we did not examine potential mechanisms underlying these associations, we can speculate from the literature that ADL performance is to a certain degree determined by cognitive component [17]. On the other hand, PA which also somewhat includes ADL has been shown to be a protective factor in the prevention of cognitive impairment and dementia [8]. However, potential effects of PA in cognitively impaired persons appear to be lower than among persons in pre-clinical stages. One study showed a day-to-day improvement in memory performance through increased physical performance, demonstrating the feasibility of the link between PA and cognition in PwD [44]. Within the CG, age emerged as a relevant predictor for decline in global cognition. This is consistent with the literature showing that age is the major known non-modifiable risk factor for dementia [45].

For classification of decliners and non-decliners in semantic fluency as well as executive function and visuospatial function, adherence to the PA intervention was a strong predictor. With regard to episodic verbal learning and memory, verbal short-term and working memory, adherence was also ranked first or second in order of significance for the classification of decliners and non-decliners. This may indicate a potential beneficial effect of the PA intervention on domain-specific cognition in our data, albeit traditional statistical analysis did not reveal statistically significant time-group effects on cognitive performance.

The tendency of higher variances observed within the IG in global cognition, as well as semantic verbal fluency, verbal short-term memory and working memory, and episodic verbal learning and memory may also suggest a potential impact of the PA intervention that was not present in the CG which only received treatment as usual. However, overall, our data using an ML-approach does not provide sufficient evidence of an impact of the PA intervention on cognitive change, or the predictability of cognitive change in PwD through adherence to the PA intervention. This is somewhat in line with the current state of research, which also shows limited, slightly positive overall effects of PA interventions on cognitive function in PwD [46].

In general, the proportion of PwD who experienced a decline in cognitive performance during the 16-week intervention was 27–48% in CG and 23–49% in IG. The distribution of decliners and non-decliners is not statistically significantly different between IG and CG. The time*group effects are also not statistically significant after the 16-week intervention with multimodal exercise combined with cognitive tasks for global cognition and for individual cognitive domains. This partly contradicts other findings, such as a meta-analysis of 18 RCTs involving 802 PwD, which found a standardized mean difference of 0.42 for high- and low-frequency interventions [46]. However, Erickson and colleagues also point out that due to the large heterogeneity of study designs, the lack of adequate description of important parameters of PA (type, amount, frequency, intensity), and the large variability of the cognitive tests used, there is at best moderate evidence for an improvement in cognitive performance with PA in PwD [47]. Even cognitive stimulation alone shows only a small short-term cognitive benefit for people with mild to moderate dementia [48]. This was found in a meta-analysis of 36 trials with very mixed results. In general, there were moderate effects on global cognition as measured by the MMSE, and the effects seemed to depend on the frequency of cognitive stimulation (twice a week or more than once a week).

### Strengths and limitations

A limitation of this study is the rather limited performance of the ML models in terms of discrimination, which limits the power and generalizability of the identified classifiers and potential predictors of cognitive decline in PwD. The reason for the generally low performance of the ML models lies to some extent in the selected variables, which, on the one hand, may not adequately predict cognitive decline, and on the other hand, the low number of variables used for classification. Further exploratory ML approaches are thus needed to derive more robust predictions, possibly by including higher-order factors (e.g., emotional distress, subjective health) and AD biomarker information [42, 43].

Another limitation is our sample size of *N* = 319, which is relatively small for the applied method and may further explain the limited performance of the ML models. Future ML-based research examining the predictive value of variables related to a PA intervention on cognitive change in PwD should thus include larger and more diverse samples.

Another limitation of our study is the lack of statistical significance, as also derived from traditional statistical analysis, with regard to potential effects of the multimodal PA intervention on cognitive function. It is conceivable that this may be explained by the design of the intervention itself, i.e., low training frequency of twice a week and implementation of the program in a group-based setting, which did not sufficiently allow for individualization, especially in terms of exercise intensity, may have prevented the intervention to elicit more effects on various outcomes of interests, including but not limited to motor and cognitive performance. However, we designed the intervention such that it fit with schedules in nursing homes (e.g., an intervention frequency of more than twice/ week would not have been feasible), and the rather low intensity was chosen to ensure safety for all participants, including those with lower motor performance levels. One more reason for the non-statistically significant effects is the range of cognitive impairment (MMSE = 10–24) within our sample as well as the different or unknown types of dementia which may have had an impact on the effectiveness of the PA intervention [49]. The wide range of cognitive abilities, coupled with unknown or mixed dementia, introduces variability that may have masked potential effects. This highlights the challenge of applying interventions in heterogeneous populations such as PwD. While the ML approach was intended to mitigate some of these issues, its effectiveness in this regard may have been limited. Future studies should consider stratifying participants by cognitive status or focusing on more homogeneous subgroups to better evaluate the impact of PA interventions on cognitive trajectory. Another reason for the lack of statistically significant effects is that the CG still received PA as part of their usual care, as is standard in many nursing homes.

Strengths of this research are the use of a ML-based approach which, to the best of our knowledge, has not been used before in examining the predictive value of variables related to a PA intervention on cognitive change in PwD. Furthermore, even though a sample size of 319 participants is rather small for ML-based research, it can be considered large for a multicenter RCT that implemented a 16-weeks PA intervention among older PwD residing in nursing homes.

## Conclusion

This exploratory ML-based analysis provided preliminary insights into the potential value of using data from a 16-week multimodal PA intervention pertaining to adherence, baseline physical performance including ADL, or other pertinent health-related variables to predict decline and non-decline in cognition in PwD residing in nursing homes. Of note, the discriminative power of ML models was low, and further analyses are needed to provide more robust results that either confirm or disconfirm our preliminary observations. Future studies should include more variables as predictors, e.g., emotional health and AD biomarkers, and a larger sample.

## Data Availability

All data and materials pertaining to this study is available upon request via the corresponding author in the form of cumulative tables. Due to the sensitivity of the data, access to the raw data is not possible.

## Abbreviations

AD: Alzheimer’s disease
ADL: Activities of daily living
AUC: Area under the ROC curve
CDT: Clock drawing test
CIRS: Cumulative illness rating scale
CG: Control group
CVLT: California verbal learning test
DS: Digit span test
EADL: Erlangen activities of daily living
FICSIT: Frailty and injuries: cooperative studies of intervention techniques–4
IG: Intervention group
ML: Machine learning
MMSE: Mini mental state examination
PA: Physical activity
PwD: Persons with dementia
PPT: Physical performance test
RCT: Randomized controlled trial
RWT: Regensburg word fluency test
STS: Sit-to-stand test
6MWT: 6-Meter walking test
TUG: Timed-up and go test
